# MRMPro: a web-based tool to improve the speed of manual calibration for multiple reaction monitoring data analysis by mass spectrometry

**DOI:** 10.1186/s12859-024-05685-x

**Published:** 2024-02-06

**Authors:** Ruimin Wang, Hengxuan Jiang, Miaoshan Lu, Junjie Tong, Shaowei An, Jinyin Wang, Changbin Yu

**Affiliations:** 1 Shandong First Medical University (SDFMU) & Central Hospital Affiliated to SDFMU, Jinan, China; 2 School of Engineering, Westlake University, 18 Shilongshan Road, Hangzhou, 310024 Zhejiang China; 3 School of Life Sciences, Westlake University, 18 Shilongshan Road, Hangzhou, 310024 Zhejiang China; 4 Institute of Advanced Technology, Westlake Institute for Advanced Study, 18 Shilongshan Road, Hangzhou, 310024 Zhejiang China; 5 Institute of Biology, Westlake Institute for Advanced Study, 18 Shilongshan Road, Hangzhou, 310024 Zhejiang China; 6 Fudan University, Shanghai, China; 7 Zhejiang University, Hangzhou, Zhejiang China; 8 College of Chemistry and Chemical Engineering, Hainan Normal University, Haikou, Hainan China; 9 Carbon Silicon (Hangzhou) Biotechnology Co., Ltd., Hangzhou, Zhejiang China

**Keywords:** MRMPro, MRM, Mass spectrometry, Batch inspection, Web service

## Abstract

**Background:**

As a gold-standard quantitative technique based on mass spectrometry, multiple reaction monitoring (MRM) has been widely used in proteomics and metabolomics. In the analysis of MRM data, as no peak picking algorithm can achieve perfect accuracy, manual inspection is necessary to correct the errors. In large cohort analysis scenarios, the time required for manual inspection is often considerable. Apart from the commercial software that comes with mass spectrometers, the open-source and free software Skyline is the most popular software for quantitative omics. However, this software is not optimized for manual inspection of hundreds of samples, the interactive experience also needs to be improved.

**Results:**

Here we introduce MRMPro, a web-based MRM data analysis platform for efficient manual inspection. MRMPro supports data analysis of MRM and schedule MRM data acquired by mass spectrometers of mainstream vendors. With the goal of improving the speed of manual inspection, we implemented a collaborative review system based on cloud architecture, allowing multiple users to review through browsers. To reduce bandwidth usage and improve data retrieval speed, we proposed a MRM data compression algorithm, which reduced data volume by more than 60% and 80% respectively compared to vendor and mzML format. To improve the efficiency of manual inspection, we proposed a retention time drift estimation algorithm based on similarity of chromatograms. The estimated retention time drifts were then used for peak alignment and automatic EIC grouping. Compared with Skyline, MRMPro has higher quantification accuracy and better manual inspection support.

**Conclusions:**

In this study, we proposed MRMPro to improve the usability of manual calibration for MRM data analysis. MRMPro is free for non-commercial use. Researchers can access MRMPro through http://mrmpro.csibio.com/. All major mass spectrometry formats (wiff, raw, mzML, etc.) can be analyzed on the platform. The final identification results can be exported to a common.xlsx format for subsequent analysis.

**Supplementary Information:**

The online version contains supplementary material available at 10.1186/s12859-024-05685-x.

## Background

Quantitative proteomics and metabolomics using LC-MS/MS has emerged as a widely adopted strategy for biomarker discovery and analysis, gaining considerable popularity over the past few decades [1]. Due to the characteristics of strong specificity, high detection sensitivity, and accurate quantification, the multiple reaction monitoring (MRM) acquisition method has emerged as the gold standard for absolute quantification [2, 3]. MRM is gaining increasing recognition and utilization in diverse fields such as pharmacokinetics and cancer research [4–6].

In the field of MRM data analysis, a variety of software solutions are available. Some MRM analysis tools are directly provided by mass spectrometer vendors, including Xcalibur by Thermo Fisher and SCIEX OS by AB Sciex. However, these software are typically limited to analyzing MRM data acquired by their respective instruments and do not support large-scale manual inspection. Others are proposed by research communities, such as MRMPROBS [7], MRMAnalyzer [8] and Skyline [9, 10]. These software tools improved the accuracy of quantification and the automation of data analysis [7, 8]. Skyline, widely regarded as the most popular software for omics data quantification, provides high-speed data analysis capabilities and comprehensive manual inspection functions. Skyline’s user-friendly visual interface significantly reduces the operational complexity of the software, thereby lowering the operating threshold for researchers.

However, automated peak picking algorithms are not always accurate, due to factors such as inadequate chromatographic separation, variations in noise, baseline and peak shape. Consequently, manual inspection becomes an essential step in MRM data analysis to enhance the accuracy and consistency. Despite various software solutions and advanced algorithms [11, 12] are available for MRM data analysis, the manual inspection process still remains a time-consuming and labor-intensive task. To handle the workload of manual inspection, numerous laboratories have implemented specialized teams solely dedicated to the inspection task. However, existing software generally lacks sufficient support for batch manual quality control, resulting in lower efficiency, especially in large cohort studies with hundreds of samples.

Here we propose MRMPro, a free and well-designed web tool focused on MRM data analysis and manual inspection in large volumes. With the goal of improving the efficiency of manual inspection, MRMPro implemented a collaborative review system based on cloud architecture, allowing multiple users to perform batch manual inspection through browsers, and provided a variety of batch operations for fast manual inspection. MRMPro supports data analysis of both MRM and schedule MRM data acquired by mass spectrometers from mainstream vendors, such as AB Sciex, Thermo Fisher, Agilent, and Waters. It mainly solves the following four problems:

1. Implementation of a standardized MRM data analysis process
2. Collaborative manual inspection capability based on web
3. An efficient compression algorithm to meet the needs of fast rendering of hundreds of chromatograms on the web side
4. An automated peak grouping algorithm to better support batch manual calibration
5. Visual components for quick manual inspection

## Implementation

### Architecture and workflow

**Fig. 1:**
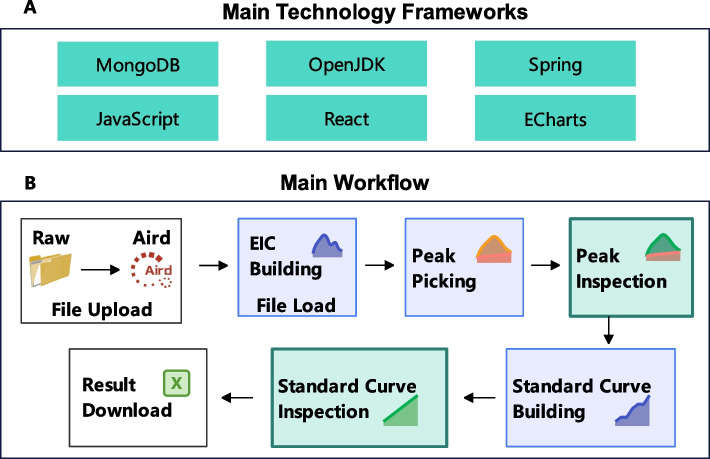
**A** The main technical frameworks used by MRMPro. **B** The main analysis workflow of MRMPro

Figure 1A shows the main technical frameworks used by MRMPro. MRMPro follows a front-end and back-end separation design approach. The back-end is developed using Java and relies on the well-established Springboot framework [13]. The front-end is built using React [14] and AntDesign [15], enabling a sophisticated and interactive user experience. To enhance chart presentation and interaction, MRMPro utilizes Echarts [16] as a presentation component. MongoDB [17] serves as the primary database container for storing project information, parameters, and analysis results. MongoDB is a high-performance, distributed database service known for its excellent performance in handling big data reading, writing, and analysis. By utilizing these open-source frameworks, MRMPro ensures transparency, extensibility, and community support, allowing for continuous improvement and customization based on evolving research needs.

MRMPro employs a classical algorithmic process based on chromatographic peak analysis for compound quantification. The workflow is depicted in Fig. 1B. Here’s an overview of the steps involved:

1. File Upload: Convert MRM mass spectrometry files with AirdPro tool to Aird format, and then upload the converted files to MRMPro. AirdPro compresses files to achieve faster upload and analysis speeds. The AirdPro conversion tool can be downloaded at https://github.com/CSi-Studio/AirdPro. The uploaded files are stored in OSS (Object Storage Service) of Alibaba Cloud.
2. File Load: When triggering data analysis, the uploaded file will be automatically downloaded into the corresponding data analysis server. The chromatograms of all transitions will then be decompressed into memory with Java AirdSDK (https://github.com/CSi-Studio/Aird-SDK).
3. Peak Picking: After performing smoothing and noise estimation on each chromatogram, MRMPro extracts all candidate peaks within retention time tolerance for each chromatogram and obtains quantitative results for transitions across all samples.
4. Peak Inspection: Perform manual inspection, which involves visually inspecting the picked peaks, their integration, and correcting any errors or inconsistencies that occurred during the automated processing to obtain the accurate relative quantification results.
5. Standard Curve Building: Input concentrations of compounds in standard samples to build standard curves for transitions. Both internal and external standard method are supported in MRMPro.
6. Standard Curve Inspection: Exclude outlier standard samples to obtain standard curves with higher R-square value. The absolute quantification results are then automatically calculated based on inspected standard curves.
7. Result Download: Download the final absolute quantification results in.xlsx format.

### Main visual interfaces

**Fig. 2:**
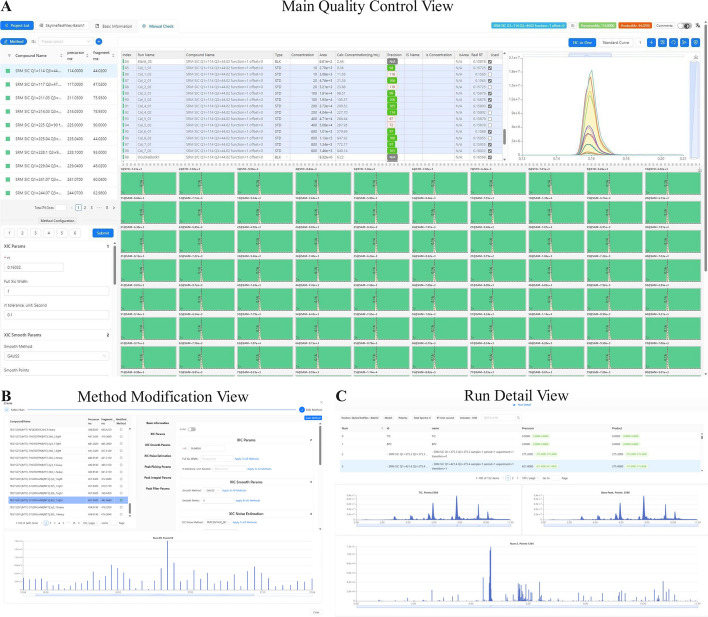
The main views of manual inspection is also the main operating panel of MRMPro. **A** The main operation panel of MRMPro includes a series of functions such as peak selection calibration, integration calibration, concentration curve calibration, identification state inspection, etc. **B** Method parameter configuration view. **C** The detailed view of every raw file. It allows researchers to view the metadata information and each chromatogram data

To improve the efficiency of manual inspection, MRMPro provides a variety of visual interfaces for users to perform fast review and calibrate. The most important interface in MRMPro is the quality control interface for fast batch manual inspection, as shown in Fig. 2A. MRMPro also provides parameter editing and sample chromatogram preview interfaces, as shown in Fig. 2B, C.

In the design of the quality control interface, we try to display as much information as possible in a limited space while ensuring the beauty, usability and rendering speed. The quality control interface consists of five parts: the transition list in the upper left, the parameter configuration panel in the lower left, the data table in the upper middle, the EIC-Matrix chart in the lower right, and the EIC-in-One chart in the upper right.

#### Transition list

The transition list shows all transitions contained in the sample, including the name of the transition, the precursor ion m/z, and the fragment ion m/z. The transition can be switched by mouse click or keyboard up, down, left, and right keys. The up and down keys are used to switch to the previous or next transition, and the left and right keys are used to switch to the transition in the same position in the previous or next page.

#### Parameter configuration panel

The parameter configuration panel shows the analysis parameters of the current transition, and different parameters can be set for different transitions. The parameters include chromatogram smoothing, noise estimation, peak picking, peak integration, peak filtering, etc., and the detailed parameters and descriptions are shown in Additional file 1: Table S1.

#### Data table

**Fig. 3:**
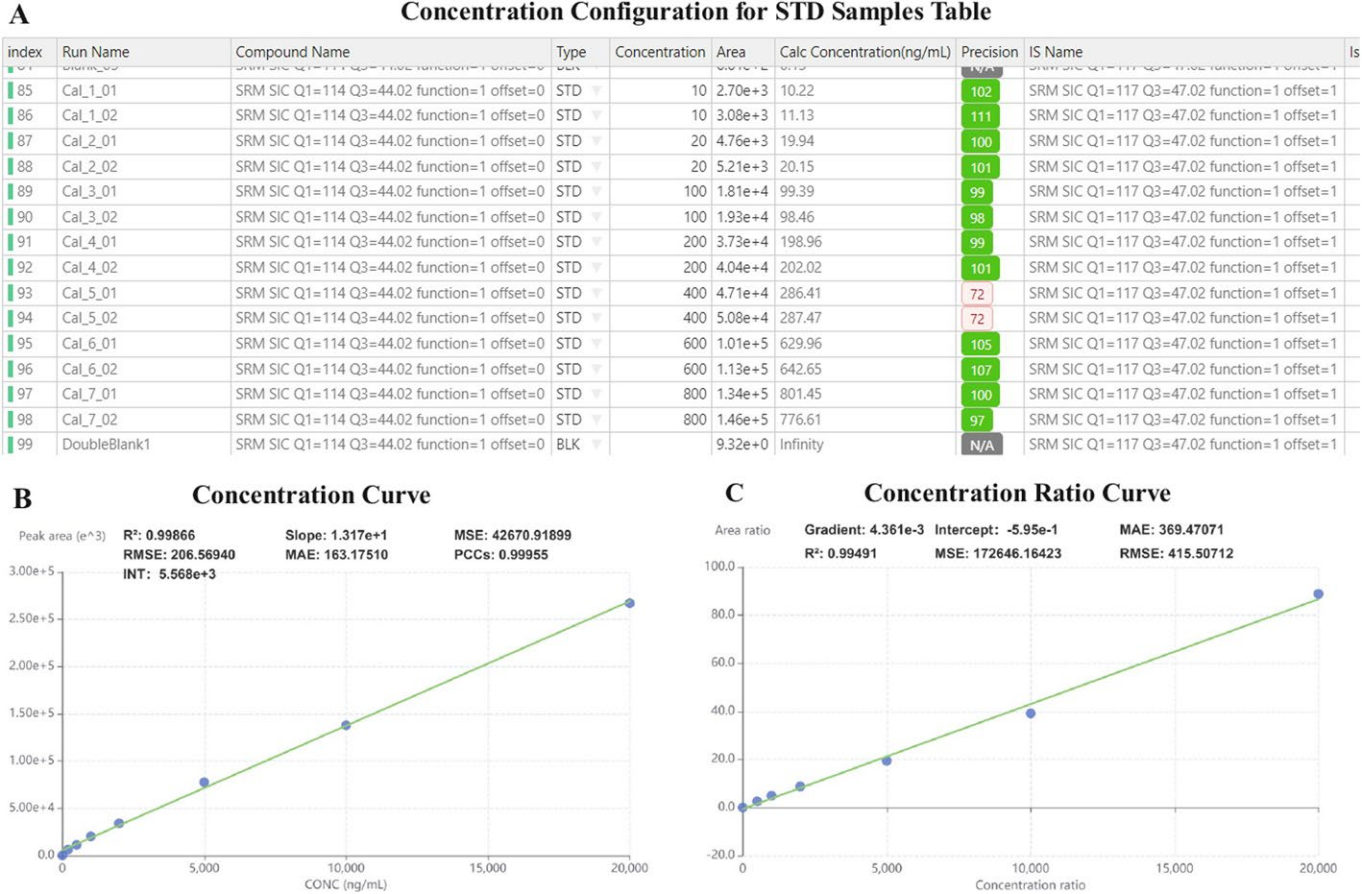
**A** Sample list. Users need to configure the concentrations of current transition in standard samples (STD) to build a standard curve. **B** Standard concentration curve. The x y coordinates correspond to the concentration value and peak area, respectively. **C** Standard concentration ration curve. The x y coordinates correspond to the concentration ratio and peak area ratio of the current transition to the selected internal standard, respectively

The data table not only shows the peak information of the current transition in all samples, but also carries important interactive functions, shown in Fig. 3A. MRMPro supports diverse quantitative strategies based on different user requirements, including relative quantification, absolute quantification with internal standard method, and absolute quantification with external standard method.

- Relative Quantification: In experiments where standard samples are not included, MRMPro can be used for relative quantification by simply extracting peak areas from chromatograms.
- External Standard Absolute Quantification: When using the external standard method, users can set the type of samples in the table, and set the concentration value of current transition in standard samples. For every transition in the standard sample, a linear mapping between the actual concentration and the peak area was created, also known as the standard curve, shown in Fig. 3B. Based on the standard curve, MRMPro can convert relative quantification results to actual concentrations for each transition.
- Internal Standard Absolute Quantification: When using the internal standard method, MRMPro supports binding an internal standard for each target transition. MRMPro normalizes the relative quantification results in different samples based on the internal standard, and then applies the normalized results to the standard curve to obtain absolute quantification results. When using the internal standard method, the calibration curve displayed shows the relative quantification ratio and the concentration ratio between the current transition and the associated internal standard transition, shown in Fig. 3C.

In standard curve building, MRMPro uses weighted least square regression to fit a linear trend-line. By normalizing the deviations with 1/*x* or $1/x^2$ weightings based on the concentration of standard samples, MRMPro can obtain more accurate fitting results. MRMPro provides five indicators to evaluate the accuracy of the constructed standard curve, including MAE, MSE, RMSE, R-square, and PCC, detailed in Appendix S1. Since transitions in standard samples are not always acquired and measured precisely, users can choose to exclude the outlier standard samples by unchecking the “Used” column in the data table, or adjust the peak areas by manual integration. MRMPro will automatically update the standard curve and indicator values, and recalculate the concentrations in all samples.

#### EIC-matrix chart

**Fig. 4:**
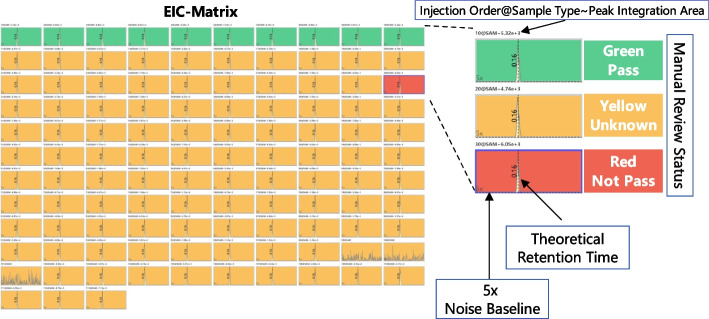
EIC Matrix chart for manual checking of identification status. Each square represents the chromatogram of a specific compound in a specific sample. Theoretical RT lines, 5x noise baselines, and quantitative peak areas are also displayed

The EIC-Matrix chart is used to display the EICs of current transition in all samples within current batch, shown in Fig. 4. It uses echarts [16] to build a high-performance chart with rich interactive capabilities. Each grid in the EIC-Matrix chart represents the EIC of current transition in a specific sample. The format of $Injection Order@Sample Type \sim Peak Integration Area$ is used for the title of each grid to show the basic information and integration result of the target compound in each sample. Injection Order is the sequence order of mass spectrometer acquisitions. MRMPro supports multiple sample types, including experimental samples (SAM), standard samples (STD), mixed samples (MIX), blank samples (BLK), etc. The theoretical RT time and fivefold noise baseline are also shown in each grid. The 5x noise baseline is a reference line with an intensity five times the maximum signal intensity in blank samples. While it is not directly utilized for peak screening, its main purpose is to provide a visual representation of the significance of the signal intensity relative to the background in each sample.

The background color of the grid represents the manual review status, green means “Pass”, yellow means “Unknown”, and red means “Not Pass”. The foreground color represents the integration area, users can efficiently obtain the quantitative area in each EIC. In manual inspection procedure, users can select single or multiple grids and annotate the manual review status with keyboard shortcuts. The keyboard shortcut “1, 2, 3” represents “Pass, Not Pass, Unknown”, respectively. After manual inspection, users can submit the results with keyboard shortcuts. The “Enter” key indicates that the transition is passed, the “Backspace” key indicates that the transition is not passed, and the “Space” key sets transition status to “Pass” and switch to the next transition. With the help of the EIC-Matrix chart, users can efficiently check the consistency of peak picking results and identification status of transitions in all samples.

#### EIC-in-one chart

**Fig. 5:**
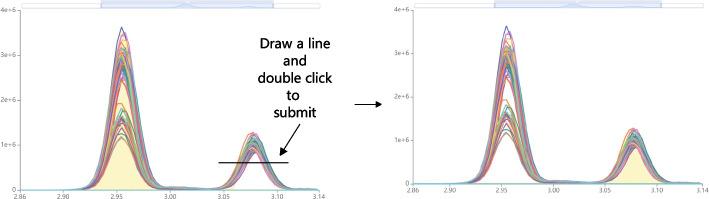
Perform batch manual integration in EIC-in-One chart. Drag a line and double-click to modify peak areas on selected EICs

The EIC-in-One is an interactive chart that superimposes selected EICs in the EIC-Matrix. The EIC-in-One chart is used for magnified visualization and manual integration of EICs from various samples. When switching between transitions, the EIC-in-One chart defaults to displaying the EICs of all samples in the current batch. Afterwards, the EIC-in-One chart will be updated with the user’s selection in the EIC-Matrix, and only superimpose the selected EICs. To change integration boundaries by manual integration, users should drag a line in the EIC-in-One chart and double-click to modify peak areas. See Fig. 5. When perform batch integration on multiple EICs, MRMPro will automatically adjust the boundaries based on the submitted integration range, analysis parameters, and the shape of EICs to ensure the accuracy of the integration. Compared with traditional methods, such as resetting target RT and drag the left and right boundaries like Skyline to adjust the integration range. The manual integration method in MRMPro is more in line with human operating habits and simplifies the operation steps.

### Analysis acceleration: aird format for MRM acquisition method

With the increase of mass spectrometer resolution and the popularity of large cohort studies, the single file size and queue size of MRM data are increasing rapidly. To reduce the bandwidth pressure and improve the reading speed, we designed a compression algorithm of MRM data for the Aird [18] format. Aird format is an open-source, high-performance mass-spectrum data compression format, has different data compression strategies for mass spectrometry data acquired by different methods. In addition to providing extremely high data compression performance to effectively reduce bandwidth costs, Aird is also a data format with extremely high read performance. In our previous paper [18], we introduced the compression strategies of the Aird format for DIA and DDA data, which compressed spectra to reduce the volume of mass spectrometry data. To improve the performance of MRMPro, in this paper, we developed a new compression strategy for MRM data, which compressed chromatograms to reduce the data volume. Users can convert vendor files to Aird format using AirdPro (https://github.com/CSi-Studio/AirdPro).

The MRM acquisition method scans and stores intensities of transitions directly as the chromatograms. The chromatogram contains two dimensions of data: retention time (RT) array and intensity array. Like the *m/z* array in spectrum data, the RT array in chromatogram data is also an incremental array. First, we save the precision of RT to the fifth decimal place(dp). When the unit of RT is minutes, its accuracy is 0.00001 min (0.0006 s). It is capable of meeting the accuracy requirements. By precision conversion, we convert RT from Double-Type to Integer-type. According to the range of positive numbers that can be expressed by the Integer-type ($0 \sim 2^{31}-1$), even when the RT time unit is second, it can express a maximum time range of about 6 h, which is substantially bigger than the usual mass-spectrum gradient time. Instead of keeping the RT directly, we store the delta values between adjacent RTs. Since the mass spectrometer’s scanning frequency is steady, the delta values between adjacent RTs should be quite small and similar, which can effectively improve the data compression rate. FastPfor [19] is then used to encode the delta data. The encoded delta data would then be compressed using the ZSTD [20] algorithm. The intensity array is compressed using Aird’s intensity compression method. We retain important metadata information from MRM mass spectrometry files in the form of a controlled vocabulary.

### Inspection acceleration: batch optimization algorithms

#### Retention time drift estimation algorithm

**Fig. 6:**
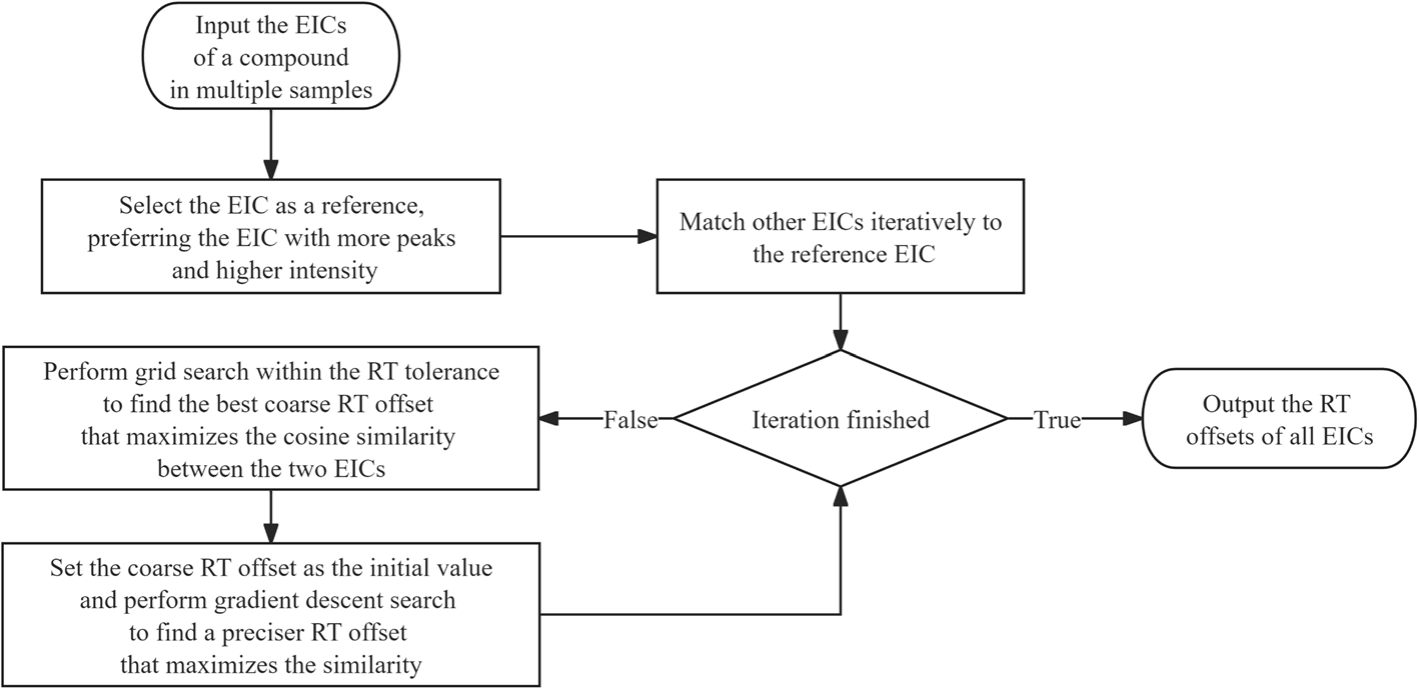
Retention time drift estimation algorithm. Estimate retention time drifts of EICs based on chromatogram similarity

Due to the instability of chromatography conditions, some transitions may have different retention time in different samples. To assess the drifts in retention time, MRMPro introduced an algorithm for estimating retention time deviations based on chromatogram similarity, shown in Fig. 6. The algorithm first selects a reference EIC with a relatively higher number of peaks and stronger intensities. The reference EIC is chosen based on the product of the number of peaks and the maximum peak height. Then, each of the remaining EICs is aligned with the reference EIC individually. This alignment process involves utilizing a combination of grid search and gradient descent methods to search for the optimal retention time deviation that maximizes the cosine similarity after calibration.

#### Peak alignment algorithm

MRMPro aligns chromatographic peaks in different samples based on the estimated retention time offsets. Firstly, initial alignment groups are established centered by retention times of peak picking results from the reference EIC. The peak picking results of other EICs are then aligned iteratively to the alignment groups. In each alignment iteration, the peak picking results are first adjusted according to the predicted retention time drifts. The peaks are then matched with the alignment groups in descending order of peak height. If the deviation falls within the full-width at half-maximum (FWHM) range of the closest group, the peak is added to the corresponding alignment group. Otherwise, create a new alignment group centered by the peak RT. In the same sample, different peaks will not be added to the same group. Within a group, the peaks represent the acquired signals of the same transition in different samples.

Once the peak alignment grouping is completed, the alignment group with the median value closest to the given target retention time is identified as corresponding to the specific transition. In cases where there are multiple chromatographic peaks with similar retention times and unstable chromatographic conditions, the peak alignment algorithm could significantly improve the consistency of peak selection results, reduce the need for manual modifications during review, and enhance manual inspection efficiency.

Based on the identification results on alignment groups, MRMPro can automatically generate initial audit statuses for each transition. For a given sample, if any of its peaks are assigned to the selected alignment group, the status is set as “Pass” and shown as green background in EIC-Matrix chart; otherwise, it is set as “Not Pass” and shown as red background in EIC-Matrix chart. By employing this approach, the time required for manual annotation of audit statuses is significantly reduced, resulting in improved efficiency of manual inspection.

#### EIC grouping algorithm

**Fig. 7:**
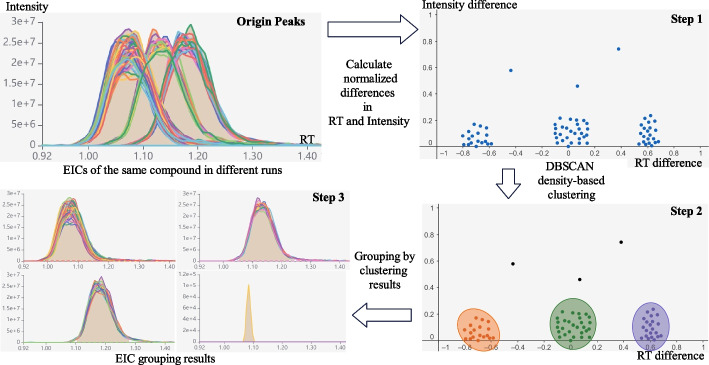
EIC grouping algorithm. Each point represents an EIC. EIC grouping algorithm assigns EICs with similar shapes to the same group and effectively distinguishes noise from actual signals

In MRM data analysis, it is common to analyze data collected from multiple days or multiple chromatographic columns simultaneously. Due to changes in chromatographic conditions, there can be significant shifts in the retention times of certain transitions between different batches, as illustrated in Fig. 7-Origin Peaks. In such cases, using the “drag-and-double-click” method for batch calibration can lead to inaccurate calibration results. It is necessary to provide an algorithm to group EICs with similar shapes and then perform batch manual integration within each group. Although MRMPro provides a manual grouping function, by dragging a square on a portion of the signal and then click the “Create new group” button to create a new group from the selected EIC. However, this manual grouping method requires complex manual operations and leads to low inspection efficiency.

To solve this problem, MRMPro also developed an automatic EIC grouping algorithm. In the first step, the algorithm calculates the normalized differences in RT and intensity of peaks in different samples. The RT differences are the drifts estimated by the retention time drift estimation algorithm. The RT and intensity differences are then normalized to the range of [-1, 1] and [0, 1]. In the second step, the Density-Based Spatial Clustering of Applications with Noise(DBSCAN) [21] algorithm is then used to cluster all points. DBSCAN is an unsupervised machine-learning clustering algorithm. Compared with the commonly used KMeans algorithm, DBSCAN does not need to determine the number of clusters and has a stable unique solution. The DBSCAN algorithm is also sensitive to outliers, which is more useful when dealing with low signal noisy peaks. In the final step, MRMPro groups EICs by DBSCAN results. As shown in Fig. 7-step.3, the EIC grouping algorithm implemented in MRMPro correctly assigns EICs with similar shapes to the same group and effectively distinguishes noise from actual signals. As an auxiliary function in the EIC-in-One chart, the EIC grouping algorithm can significantly improve the efficiency of manual inspection.

## Results

### Evaluations of compressed data formats

#### Test datasets

Three public MRM datasets were used in evaluation, which are the Skyline test dataset, the PXD031038 dataset, and the PXD009543 dataset, containing 113 files, 18 files, and 24 files, respectively. The Skyline test dataset is the test dataset provided by Skyline. The other two datasets can be downloaded from ProteomeXchange [22] with the accession numbers PXD031038 and PXD009543. The download links are shown in Table 1.

**Table 1:** Datasets address

Datasets	Address
Skyline test files	https://skyline.ms/tutorials/SmallMoleculeQuantification.zip
PXD031038	https://proteomecentral.proteomexchange.org/cgi/GetDataset?ID=PXD031038
PXD009543	https://proteomecentral.proteomexchange.org/cgi/GetDataset?ID=PXD009543

#### Compression performance

**Fig. 8:**
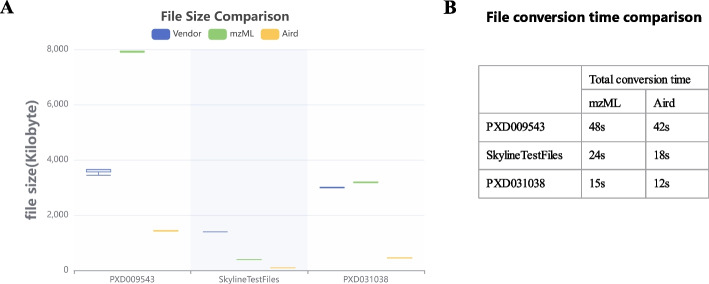
**A** File size comparison of the vendor, mzML, and Aird formats on three evaluation datasets. **B** Compression time comparison of the mzML format and the Aird format

We converted the vendor files to mzML format (32-bit) and Aird format using MSConvert [23] and AirdPro [18], respectively. In comparison of file volumes of different data formats, the file volume of Aird format has the highest compression rate and is much smaller than the mzML format and vendor formats, shown in Fig. 8A. In the PXD009543 dataset, Aird format is only 40% of Vendor format and 18% of mzML format. In the test dataset from Skyline, the Aird format is only 5% of the Vendor format and 20% of the mzML format. In the PXD031038 dataset, Aird format is only 15% of Vendor format and 14% of mzML format. As a result, using the Aird format for data uploaded to MRMPro server can significantly lower bandwidth costs and improve cross-platform data processing. AirdPro (version 5.1 or later) supports conversion for the MRM acquisition method and is available at https://github.com/CSi-Studio/AirdPro.

The compression time of the mzML format and the Aird format is shown in Fig. 8B. The compression time of the Aird format is slightly shorter than that of the mzML format. The Aird format achieved competitive compression time while ensuring the compression ratio.

#### Reading performance

MRM data reading typically falls into the following two categories: One is to read and decode all chromatograms from the original file into memory. This includes the reading of metadata and the decoding of all chromatograms, which is usually done when the data is read for the first time. The other is to decode a target chromatogram into memory. This is usually done by manual review or when new parameters are applied to existing chromatographic peaks. One difference between the two is that the former requires complete reading of all index information, which consumes a lot of time. The latter needs to read target chromatograms in dozens or even hundreds of samples, which has certain requirements for the random reading ability of chromatographic data. Because the internal structure of the vendor files is not available, and the SDK is not open source, we cannot accurately measure how quickly each vendor file is fully decoded into memory. Here we only compare the full read speed of the most common mzML format with that of the Aird format. The Aird file reading code in MRMPro was written in Java. To ensure consistency and eliminate any performance variations caused by programming languages or the implementation of mzML file reading code, we directly utilized the well-established and mature mzML file reading code from MZmine3 [24], which was also written in Java.

**Fig. 9:**
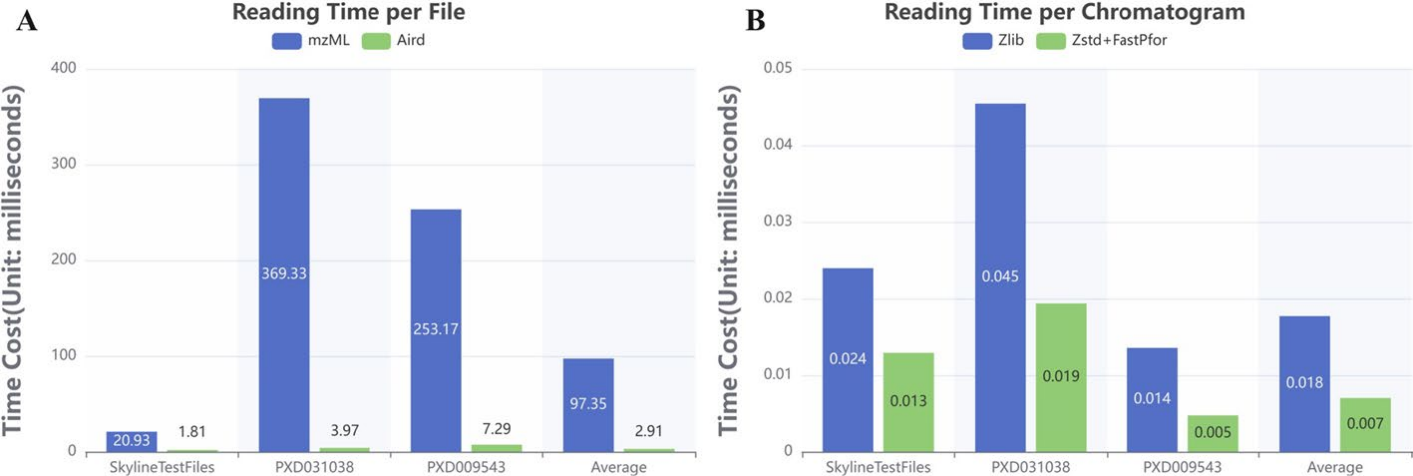
**A** Average reading time of full MRM file. **B** Average reading time of a single chromatogram

The reading speed of the mzML format and the Aird format is shown in Fig. 9. In comparison on full file reading speed, the Aird format has a faster full read speed on all three datasets, shown in Fig. 9A. The Aird format is about 33 times faster than the mzML format on average, due to the higher compression ratio and the use of JSON-encoded metadata. In comparison on single chromatogram reading speed, which is more important for recalculations in inspection, the Aird format is about 2.5 times faster than the mzML format on average, shown in Fig. 9B. The Zstd and FastPfor strategy used by the Aird format showed higher reading performance than the Zlib strategy used by the mzML format. The above tests were performed on a desktop computer with Intel i9-12gen CPU, 128GB memory and 512GB SSD disk. In general, using the Aird format with extended support for MRM acquisition method can effectively reduce the mass-spectrum file size, thus effectively reducing the bandwidth and memory requirements in web-oriented scenarios.

### Evaluations of quantification performance

Skyline is the most popular software in MRM data analysis. We compared MRMPro with Skyline on the Skyline test dataset. Based on the recommended analysis parameters from Skyline’s tutorial, we compared the quantitative results of two transitions, Drug_light and Drug_heavy, across 113 test files.

**Fig. 10:**
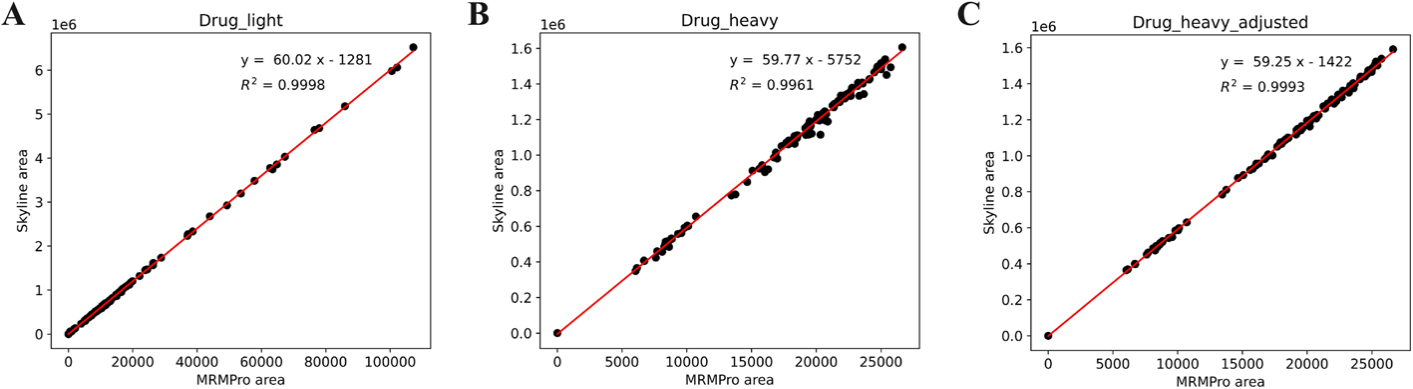
**A** MRMPro versus Skyline quantification results of Drug_light. **B** MRMPro versus Skyline quantification results of Drug_heavy. **C** MRMPro versus Skyline(inspected) results of Drug_heavy

**Fig. 11:**
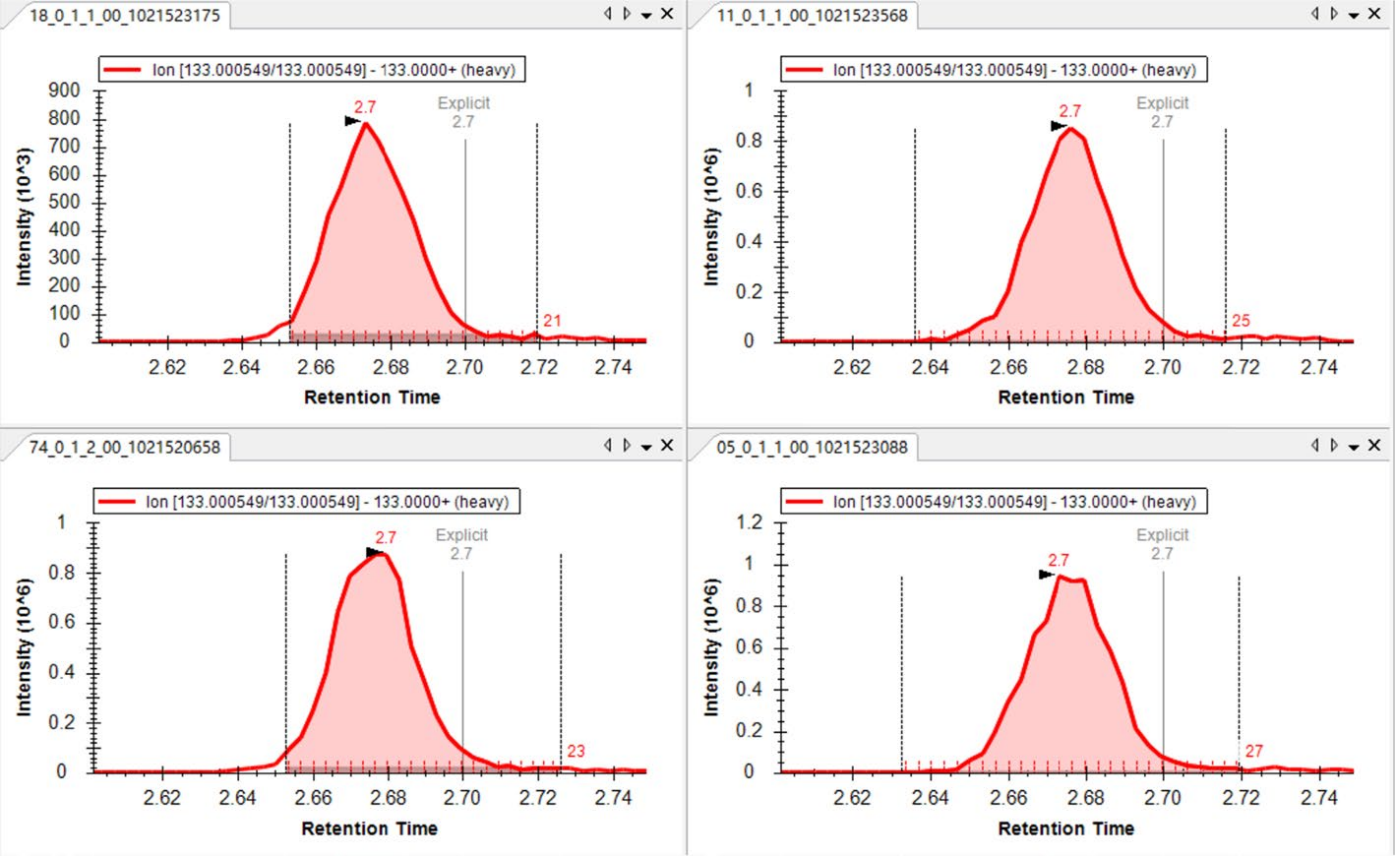
Baseline determination errors in Skyline results

Firstly, we conducted a comparison of the algorithm-generated results for their consistency, as illustrated in Fig. 10A, B. In the comparison of Drug_light results, we observed a strong correlation between the quantification results obtained by MRMPro and Skyline, with an R-square value of 0.9998. However, in the comparison of Drug-heavy results, the R-square value decreased to 0.9961, and we noticed that in some samples, MRMPro exhibited relatively higher quantification results compared to Skyline. We attempted to identify the source of the errors and found errors in the baseline determination in Skyline results, as depicted in Fig. 11. Skyline estimates baseline intensity by selecting the lowest intensity at peak boundaries, which cannot describe background noises accurately. When using the gradient truncation method to determine peak boundaries, Skyline’s baseline estimation results are noticeably higher, leading to lower quantification results. Meanwhile, MRMPro estimates baseline intensity on each side of the peak separately and assumes the baseline intensity change is linear inside the peak. The baseline intensity of each side is estimated by selecting the lowest intensity within user defined RT tolerance outside the peak. The baseline estimation method in MRMPro is more accurate and robust. No errors were observed in the quantification results obtained by MRMPro.

Then, we performed manual integration of the Skyline results for Drug-heavy and compared them with the initial results obtained by MRMPro, as illustrated in Fig. 10C. The manually integrated results of Skyline exhibited a strong correlation with the results obtained by MRMPro, with an R-square value of 0.9993. This finding confirms the superior accuracy of quantification achieved by MRMPro in comparison to Skyline.

### Evaluations of manual inspection performance

**Fig. 12:**
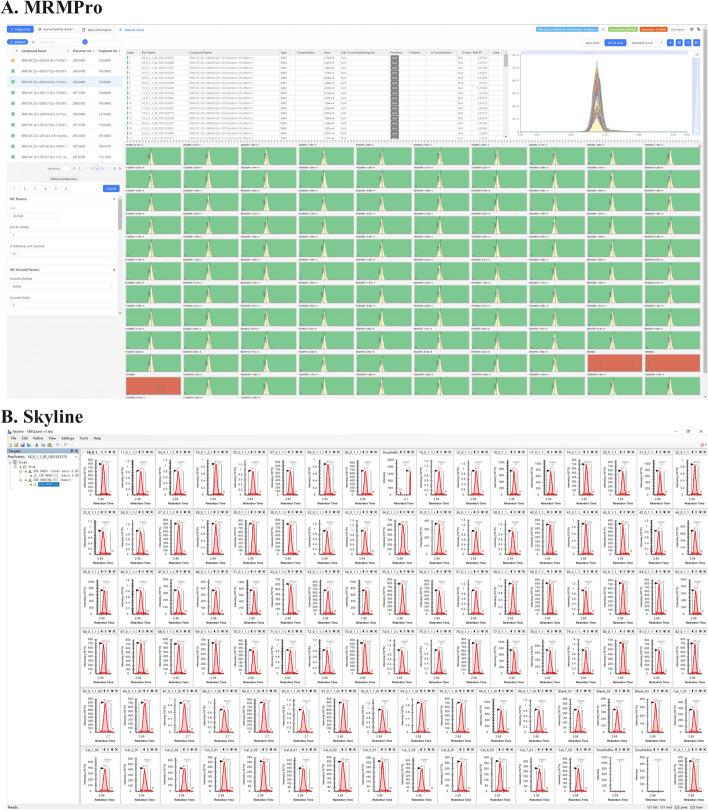
The inspection pages of MRMPro and Skyline

The efficiency of manual inspection is influenced by various factors, including dataset distributions and experience with different software, making it difficult to reliably measure the improvement in audit performance. Here, we compared the inspection interfaces of MRMPro and Skyline to evaluate the efficiency of manual inspection indirectly, as shown in Fig. 12. We utilized the same 32-inch display to evaluate the interfaces. To optimize the visual experience of Skyline, non-essential display elements such as legends were disabled, the font size was minimized, and peak shapes were scaled to a visible extent.

In the preview of the EICs, the MRMPro interface clearly displayed the target peaks of 110 samples along with their surrounding chromatographic profiles. It also exhibited distinct visualization of integration boundaries and baselines. For the portion of data that exceeds the screen capacity, MRMPro supports using mouse scroll wheel to navigate through the remaining peaks smoothly.

Skyline has the same matrix display mode by arranging the graphs in tiled mode, it could display the target peaks in 100 samples. However, in order to clearly observe the target peaks, users have to zoom in the peak shape, which will cause the surrounding signal distribution to be unable to be displayed. Additionally, the display quality of integration boundaries and baselines was inferior to that of MRMPro. As for the portion of data that exceeds the screen capacity, Skyline lacks a paging function, requiring manual opening of individual samples by cross-referencing with the sample list. Furthermore, in Skyline, when opening a sample that exceeds the visualization capacity of the screen, it occupied a window and covers the previously displayed EIC.

MRMPro offered more information on its interface. For instance, the parameter panel in the bottom left corner facilitated quick adjustment of parameters to recalculate peak picking results for the current transition. The interactive data table in the center top provided easy access to view integration areas, adjust sample types, configure standard sample concentrations, and determine their inclusion in generating standard curves. The EIC-in-One graph in the top right corner offered convenient features such as batch integration through “drag-and-double-click” and automatic grouping.

Although it is challenging to quantitatively measure the efficiency improvement of MRMPro’s inspection interface over Skyline, the design and richness of functionalities in MRMPro’s inspection interface suggest higher efficiency.

## Conclusions

With the increasing popularity of untargeted data analysis, high-throughput mass spectrometry technology has developed rapidly. As the most important mass spectrum acquisition methods, Data dependent acquisition(DDA) and data independent acquisition(DIA) are crucial at the stage of large-scale target filtering. However, no matter what high-throughput acquisition method is used for target filtering, it is a reliable methodology to use MRM acquisition method to confirm the selected targets. Since MRM usually represents the final confirmation of a molecular target, manual inspection is necessary in most scenarios. However, as the sample queue continues to grow, the cost of a manual inspection becomes higher and higher. Although many algorithms or methodologies have emerged to reduce the cost of manual inspection, these methods or algorithms often carry additional experimental costs, or are not common scenarios. As the first tool to focus on how to improve the efficiency of batch manual inspection for MRM data analysis, MRMPro discusses and implements efficient manual inspection methods in detail from the perspective of collaborative inspection, high-performance transmission and calculation, batch operation, etc. This concept has also been generalized and implemented in untargeted metabolomics data analysis [25]. Currently, MRMPro is dedicated to improving the efficiency of manual inspection. Despite demonstrating higher quantification accuracy compared to Skyline, MRMPro is built upon systematic optimizations of traditional algorithms and relies on classical algorithms to provide a stable and precise analytical framework. Moving forward, we plan to incorporate more advanced analytical algorithms to improve the accuracy of peak extraction and further enhance the efficiency of manual inspection.

### Supplementary Information

**Additional file 1. Table S1.** Analysis parameters and explanations; **Appendix S1.** Indicators for evaluating the goodness of standard curve fitting.

## Data Availability

Project name: MRMPro. License: Not opensource but free to use for non-commercial. User Manual: https://github.com/CSi-Studio/MRMPro-Doc. MRMPro Online service: http://mrmpro.csibio.com. AirdPro: https://github.com/CSi-Studio/AirdPro. AirdSDK: https://github.com/CSi-Studio/Aird-SDK. Test Datasets: Skyline Test Files (https://skyline.ms/tutorials/SmallMoleculeQuantification.zip); PXD031038 (https://proteomecentral.proteomexchange.org/cgi/GetDataset?ID=PXD031038); PXD009543 (https://proteomecentral.proteomexchange.org/cgi/GetDataset?ID=PXD009543). Restrictions to use by non-academics: Commercial authorization required. Supplementary File: MRMPro_bmc_bioinformatics_supplementary_R3.pdf.

## Abbreviations

RT: Retention Time
MS: Mass Spectrometry
dp: Decimal place
LC: Liquid Chromatograph
QC: Quality Control
QA: Quality Assurance
LTR: Long-Term Reference
SOP: Standard Operating Procedure
SIMD: Single Instruction Multiple Data
DBSCAN: Density-Based Spatial Clustering of Applications with Noise
DDA: Data dependent acquisition
DIA: Data independent acquisition
