# Supplementary material for: MRMPro: a web-based tool to improve the speed of manual calibration for multiple reaction monitoring data analysis by mass spectrometry
Source: BMC Bioinformatics. 2024 Feb 6;25:60. doi: 10.1186/s12859-024-05685-x (PMC10848457; doi:10.1186/s12859-024-05685-x)
Supplement: Supplementary file 1 — Additional file 1. Table S1. Analysis parameters and explanations; Appendix S1. Indicators for evaluating the goodness of standard curve fitting. [file 12859_2024_5685_MOESM1_ESM.pdf]

Supplementary Information

MRMPro: A web-based tool to improve the speed of manual calibration for multiple reaction monitoring data analysis by mass spectrometry

Ruimin Wang, Hengxuan Jiang, Miaoshan Lu, Junjie Tong, Shaowei An, Jinyin Wang and Changbin Yu

Table S1. Analysis parameters and explanations.

Workflow	Parameter name	Example value	Explanation
EIC Extraction	FullEicWidth	0.1	Extract EIC with RT width of 0.1 min
	minMzTolerance	0.001	If calculated M/z ppm tolerance.is smaller than 0.001Da, extract EIC with a ±0.001Da M/z window
	MzTolerance	10.0 ppm	Extract EIC with a ±10ppm M/z window
		0.05 Da	Extract EIC with a ±0.05Da M/z window
	RtTolerance	0.5	Find target peaks in ±0.2min of library RT
EIC Smoothing	SmoothMethod	LINEAR	Smoothing with average intensity
		GAUSS	Smoothing with Gaussian-distributed weight
		SAVITZKY_GOLAY	Smoothing with Mexican-hat weight
		PROPRO_GAUSS	Smoothing by Gaussian regression weight
		NONE	Do not use smoothing
	SmoothPoints	5	5 point wide smooth window (center point, left 2 points and right 2 points; must be an odd number)
EIC Noise Estimation	EicNoiseEstimation	PROPRO_EIC	Estimate noise by signal local distribution
		AMPLITUDE_EIC	Bin all intensities in EIC with NoiseAmplitude. Consider the intensity of the highest frequency bin as noise intensity
		PERCENTAGE_EIC	Sort all intensities in EIC. Consider NoisePercentage percentile intensity as noise intensity
	NoiseAmplitude	100	Set the noise intensity amplitude width at 100

	NoisePercentage	20	Consider the 20% lowest intensity of the current EIC as the noise threshold
Peak Selection	PeakFindingMethod	PROPRO	Self-developed peak selection algorithm, used for smooth peak separation
		WAVELET	Maxican-hat regression, reproducted from XCMS,MZmine2, used for noisy peak selection
		LOCAL_MINIMUM	Analyze the first and second derivatives, reproducted from MZmine2, used for narrow peak selection
Peak Integration	BaselineMethod	TOLERANCE	Consider the minimum intensity with BaselineRtTolerance
		NONE	Not use baseline detection
	BaselineRtTolerance	0.05	The RT width of baseline detection out of each side of the peak boundary
	FirstDerivativeCutoff	0.1	Set peak boundary at 0.1 times of max first derivative of each side
Peak Screening	PeakNoiseEstimation	SLIDING_WINDOW_PEAK	Estimate peak noise by sliding window out of peak boundary
		WAVELET_COEFF_PEAK	Estimate peak noise by Wavelet peak detection fitting performance
	StnThreshold	1	Minimum signal to noise ratio
	minPeakHeight	3000	Excluding peaks with apex intensity less than 3000
	minPeakWidth	0.02	Excluding peaks with RT width less than 0.02min
	minPeakPoints	7	Excluding peaks with less than 7 points
	maxNoiseRatio	0.5	Excluding peaks with noise signal ratio higher than 0.5
	minObviousness	0.7	Excluding peaks with obviousness less than 0.7

Appendix S1. Indicators for evaluating the goodness of standard curve fitting.

1. Mean Absolute Error (MAE):

$$MAE = \frac{1}{n}\sum_{i=1}^{n} |y_i - \hat{y}_i|$$

The MAE indicates the average degree of deviation between the observed and predicted values. A smaller MAE indicates that the model's average prediction error is smaller, indicating that the model has improved predictive ability.

2. Mean Square Error (MSE):

$$MSE = \frac{1}{n}\sum_{i=1}^{n} (y_i - \hat{y}_i)^2$$

The MSE gives the average degree of difference between the predicted value and the actual observed value, and the square of the difference is amplified. A smaller MSE indicates that the average prediction error of the model is smaller, which indicates the prediction ability of the model is better.

3. Root Mean Square Error (RMSE):

$$RMSE = \sqrt{\frac{1}{n}\sum_{i=1}^{n} (y_i - \hat{y}_i)^2}$$

The RMSE represents the average degree of deviation between the predicted value and the actual observed value. It squares the MSE to account for the extent of the error in the same units as the original dependent variable. The lower the RMSE, the lower the average prediction error of the model, and thus the better the model's predictive ability.

4. R Square (R-square):

$$R^2 = 1 - \frac{\sum_i (y_i - \hat{y}_i)^2/n}{\sum_i (y_i - \bar{y})^2/n} = 1 - \frac{RMSE}{\mathrm{Var}}$$

R^2 is a measure of how well a regression model fits observational data. It represents the percentage of the dependent variable's variance that the independent variable can account for, with values ranging from 0 to 1. The closer R^2 is to 1, the better the model can explain the observed data's variability and the greater the fitting effect. The closer R^2 is to 0, the more it indicates that the model cannot adequately explain the variability of the observed data and that the effect of fitting is inadequate.

5. Pearson Correlation Coefficient:

$$r = \frac{\sum_{i=1}^{n} (x_i - \bar{x})(y_i - \bar{y})}{\sqrt{\sum_{i=1}^{n} (x_i - \bar{x})^2}\sqrt{\sum_{i=1}^{n} (y_i - \bar{y})^2}}$$

In this equation, x_i and y_i represent the observed values of the two variables, $\bar{x}$ and $\bar{y}$ represent the mean values of the corresponding variables The Pearson correlation coefficient reflects the strength and direction of the linear relationship between two variables. When the correlation coefficient approaches 1, it indicates that there is a strong positive correlation between the two variables. When the correlation coefficient approaches -1, it indicates that there is a strong negative correlation between the two variables. When the correlation coefficient is close to 0, it means that the linear relationship between the two variables is weak or that no linear relationship exists.
